# Correction: Ahn et al. Innovative qPCR Algorithm Using Platelet-Derived RNA for High-Specificity and Cost-Effective Ovarian Cancer Detection. *Cancers* 2025, *17*, 1251

**DOI:** 10.3390/cancers18061033

**Published:** 2026-03-23

**Authors:** Eunyong Ahn, Se Ik Kim, Sungmin Park, Sarah Kim, Hyunjung Kim, Hyejin Lee, Heeyeon Kim, Eun Ji Song, Kyung-Ah Hwang, TaeJin Ahn, Yong-Sang Song

**Affiliations:** 1 Foretell My Health, Inc., 558 Handong-ro Buk-gu, Pohang 37554, Republic of Koreaeunjisong@foretell.bio (E.J.S.); 2 Department of Obstetrics and Gynecology, Seoul National University College of Medicine, Seoul 03080, Republic of Korea; seikky@naver.com; 3 Cancer Research Institute, Seoul National University College of Medicine, Seoul 03080, Republic of Korea; 4 Department of Genomic Strategy, Samkwang Labtree, Seoul 06740, Republic of Korea; 5 Department of Advanced Convergence, Handong Global University, Pohang 37554, Republic of Korea; 6 Department of Obstetrics and Gynecology, Myongji Hospital, Goyang 10475, Republic of Korea

**Addition of an Author**

Kyung-Ah Hwang was not included as an author in the original publication [1]. The corrected Author Contributions statement appears here. The authors state that the scientific conclusions are unaffected. This correction was approved by the Academic Editor. The original publication has also been updated. Dr. Kyung-Ah Hwang designed and conducted PCR-based marker identification experiments related to ovarian cancer, participated in primer design and optimization, contributed to data analysis and interpretation, and provided critical intellectual input through manuscript review and revision.

**Addition of an Affiliation**

Department of Genomic Strategy, Samkwang Labtree, Seoul 06740, Republic of Korea

**Conflicts of Interest**

Eunyong Ahn, Se Ik Kim, Sungmin Park, Sarah Kim, TaeJin Ahn, and Yong-Sang Song are stockholders of Foretell My Health, Inc. Eunyong Ahn, Sungmin Park, Sarah Kim, Hyunjung Kim, Hyejin Lee, Eun Ji Song, TaeJin Ahn are affiliated with the company Foretell My Health, Inc. Kyung-Ah Hwang is affiliated with the company Samkwang Labtree. We affirm that this conflict of interest does not affect the integrity, objectivity, or validity of the research conducted. All efforts have been made to ensure that the research is conducted impartially and accurately. Patent applications related to some parts of these findings in this study have been filed by the authors.

**Author Contributions**

E.A., S.I.K., T.A. and Y.-S.S. designed and supervised the research. S.P. and S.K. organize clinical study and lead computational methodology development. S.P. and S.K. performed Bioinformatic analysis. H.L., H.K. (Heeyeon Kim) and H.K. (Hyunjung Kim) performed experiments. S.I.K. and Y.-S.S. recruited patients for clinical research. E.A. wrote the first version of the manuscript. E.J.S. edited the manuscript and wrote the final version of manuscript. K.-A.H. designed and conducted PCR-based marker identification experiments related to ovarian cancer; participated in primer design and optimization; contributed to data analysis and interpretation. All authors have read and agreed to the published version of the manuscript.
